# Ablation efficiency and relative thermal confinement measurements using wavelengths 1,064, 1,320, and 1,444 nm for laser-assisted lipolysis

**DOI:** 10.1007/s10103-012-1100-9

**Published:** 2012-04-26

**Authors:** Jong-In Youn, J. David Holcomb

**Affiliations:** 1 Department of Biomedical Engineering, College of Medical Science, Catholic University of Daegu, Gyeongbuk, South Korea; 2 Holcomb Facial Plastic Surgery and Institute for Integrated Aesthetics, 1 S. School Avenue, Ste 800, Sarasota, FL 34237 USA

**Keywords:** Ablation, Ablation crater, Ablation efficiency, Fat, Laser lipolysis, Mass loss, Thermal confinement, Wavelength dependence

## Abstract

Laser-assisted lipolysis is routinely used for contouring the body and the neck while modifications of the technique have recently been advocated for facial contouring. In this study, wavelength-dependence measurements of laser lipolysis effect were performed using different lasers at 1,064, 1,320, and 1,444 nm wavelengths that are currently used clinically. Fresh porcine skin with fatty tissue was used for the experiments with radiant exposure of 5–8 W with the same parameters (beam diameter = 600 μm, peak power = 200 mJ, and pulse rate = 40 Hz) for 1,064, 1,320 and 1,444 nm laser wavelengths. After laser irradiation, ablation crater depth and width and tissue mass loss were measured using spectral optical coherence tomography and a micro-analytical balance, respectively. In addition, thermal temporal monitoring was performed with a thermal imaging camera placed over ex vivo porcine fat tissue; temperature changes were recorded for each wavelength. This study demonstrated greatest ablation crater depth and width and mass removal in fatty tissue at the 1,444 nm wavelength followed by, in order, 1,320 and 1,064 nm. In the evaluation of heat distribution at different wavelengths, reduced heat diffusion was observed at 1,444 nm. The ablation efficiency was found to be dependent upon wavelength, and the 1,444 nm wavelength was found to provide both the highest efficiency for fatty tissue ablation and the greatest thermal confinement.

## Introduction

Neodymium-doped lasers are widely used in medicine across a range of specialties and with specific applications requiring substantially different thermal effects. Significant variations in laser tissue interaction are enabled via wavelength selection and optical and other properties of the targeted and immediately adjacent tissues (e.g., ability to selectively absorb light and ability to withstand collateral thermal damage) and treatment mode (e.g., continuous wave versus pulsed and contact versus non-contact) [1]. Neodymium-doped laser radiant energy for non-surgical, non-contact applications penetrates skin tissue up to several millimeters and selectively targets discrete structures or substances (selective photothermolysis) to produce primarily coagulative tissue effects [1]. In contrast, surgical applications of neodymium-doped lasers more typically involve contact mode and primarily generate photoablative and, to a lesser extent, photocoagulative changes that are relatively superficial (e.g., up to 600 μm and an additional 50–100 um, respectively) [1].

Laser lipolysis is a popular surgical application of neodymium-doped lasers but for which tissue interaction is insufficiently characterized. Different wavelengths may vary in effectiveness targeting substances present in the subcutaneous lasing microenvironment, including collagen (e.g., adipocyte membranes; e.g., fibrous septae), fat (i.e., adipocyte lipid content), hemoglobin, and water (e.g., within fibrous and vascular structures; e.g., exogenous water content introduced with infiltration of local anesthetic or tumescent solution).

Possible mechanisms for immediate and delayed effects of laser lipolysis include direct photoablation and photocoagulation (less wavelength-dependent, direct heating of tissue in close proximity to fiber tip), selective photothermolysis (highly wavelength-dependent, possibly also involving photoacoustic or photodisruptive effect), photomechanical ablation effect (resulting from pulsed laser energy discharge with non-linear explosive tissue interaction at tip of fiber), activity of liberated adipocyte lipases, and microcirculation thrombosis [2–5]. While the relative importance of these putative mechanisms remains under investigation, laser lipolysis must be substantially affected by wavelength and the optical properties of subcutaneous fat.

**Fig. 1:**
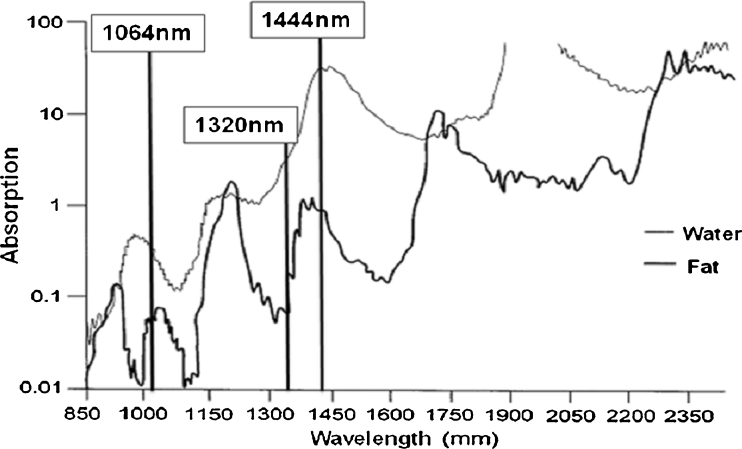
Absorption coefficient (logarithmic, *Y*-axis) versus wavelength dependence (linear, *X*-axis) for water and fat [3]

Evaluation of fat and water absorption for neodymium-doped laser wavelengths used in first generation (lower power, single wavelength) and second generation (higher power, dual wavelength) lipolysis lasers reveals low (1,064 nm) and only slightly higher (1,320 nm) absorption coefficients for fat and water on a logarithmic scale (Fig. 1) [3,6]. A recent study suggests that 1,064 and 1,320 nm lipolysis lasers do not rely on conventional laser tissue interactions (where wavelength specificity should ideally enable selective photothermolysis of fat, the primary tissue target) and that collagen or water within fibrous tissue septae is preferentially damaged by these wavelengths with injury to adjacent adipocytes occurring secondarily [3].

An ideal wavelength for laser lipolysis is highly selective for the intended tissue target(s) and also has an energy delivery or thermal profile that is highly controlled such that the effects of the absorbed energy are very predictable. Thermal confinement and thermal diffusivity are opposing characteristics of fiber laser tissue interaction that are of previously unrecognized importance with regard to wavelength selection and energy delivery during laser lipolysis. Large volume treatment areas (e.g., body contouring), addition of relatively large amounts of exogenous water (tumescent solution) to the treatment areas, and temperature elevation endpoint monitoring retarded discussion of thermal confinement. Development of a new laser lipolysis application for facial contouring with comparatively very small volume treatment areas and with immediately proximate delicate anatomic structures raises the specter of unintended collateral thermal damage and the need for relative thermal confinement.

The micropulsed (100 μs) 1,444 nm neodymium-doped lipolysis laser (AccuSculpt™, Lutronic Inc., San Jose, CA, USA) was optimized to enable selective photothermolysis of subcutaneous fat. Evaluation of optical absorption spectra for fat and water at 1,444 nm reveals unique nearly convergent absorption peaks (Fig. 1) [3]. In addition, a minor (anhydrous) collagen absorption peak is present in this region [7]. Light absorption at 1,444 nm is approximately one order of magnitude greater for fat and greater by more than ten orders of magnitude for water versus wavelengths (1,064 and 1,320 nm) used in first and second generation lipolysis lasers (Fig. 1) [6]; these differences infer substantial variation in laser tissue interaction among neodymium-doped lipolysis lasers.

While an in vivo animal study has already demonstrated a greater lipolytic effect of the 1,444 nm versus 1,064 nm lipolysis lasers, the 1,320 nm lipolysis laser was not evaluated [8]. We believe that additional ex vivo data regarding fatty tissue ablation efficiency and thermal diffusion characteristics among these three wavelengths will further safest practices with clinical use of neodymium-doped lipolysis lasers (e.g., facial contouring). We present data herein regarding the thermal temporal nature and the optical mechanical effects (adipose tissue ablation efficiency and optical coherence tomography) of lipolysis laser tissue interaction. In this context, we compare optical and thermal properties of neodymium-doped lipolysis lasers of three different wavelengths (1,064, 1,320, and 1,444 nm) and review related implications for lipolysis laser tissue interaction.

## Materials and methods

### Fat tissue harvest

Fresh porcine abdominal skin with fat tissue was obtained from a local abattoir. The fat tissue was obtained 2 hours postmortem. The prepared porcine tissue was placed on a table, and the skin was removed using a scalpel. The fat tissue was cut into squares measuring approximately 3 × 3 × 3 cm, and the specimens were then stored in a physiologic saline solution just prior to the experiment.

### Crater depth and width measurements with OCT

Three different lasers, with wavelengths (*λ*) of 1,064, 1,320, and 1,444 nm were used in the experiments. The 1,064 and 1,320 nm lasers were developed by Lutronic Corp., South Korea for research purposes. The 1,444 nm laser is a commercial laser (AccuSculpt™) also developed by Lutronic Corp., South Korea. Laser irradiation of porcine tissue was delivered by a 600-μm silica multimode fiber with individual energy outputs for the three different lasers set to predetermined values and confirmed using an energy meter (PM320E, Thorlabs, Inc., Newton, NJ, USA). Ten samples were irradiated for the measurements at each laser wavelength per parameter set.

**Fig. 2:**
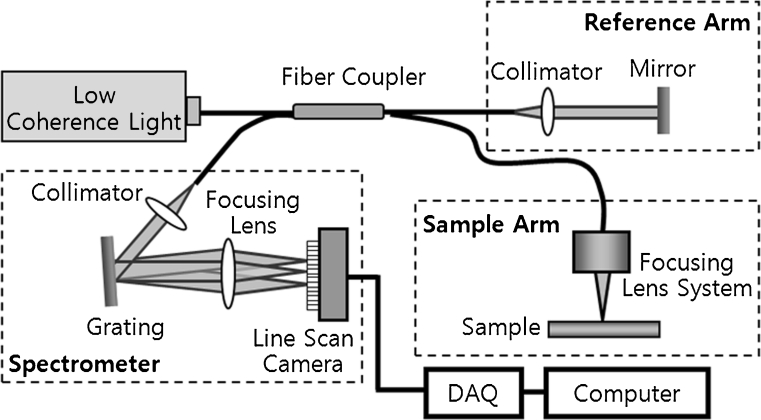
Schematic of spectral domain optical coherence tomography (OCT) using Fourier domain detection (spectral resolution, 12 μm; maximum scan depth, 3.0 mm; and maximum scan length, 6 mm)

Optical coherence tomography (OCT) is a noninvasive imaging technology that uses light reflected from within tissue to generate two-dimensional sectional images of the tissue structure in a manner similar to but with higher resolution than is obtained with ultrasound. OCT is reported to provide images that correlate well with histology at a sufficient resolution to identify morphologic changes within the tissue [9–12]. To investigate the crater depth and width of the fat tissue after laser irradiation, a spectral domain OCT system was used (Fig. 2). Low-coherence light having a center wavelength of *λ* = 840 nm with a full-width half-maximum bandwidth of 50 nm was coupled into the source arm of a fiber-based Michelson interferometer. Back-reflected lights from the reference and sample arms were guided into a spectrometer. The detected signal was then image processed by a computer. In this system, an imaging depth was 3.2 mm in air, and the acquired OCT images from the sample were 6 × 1.6 mm. The OCT images of the craters after each laser irradiation at 5–8 W were explored and taken at the deepest position in the crater region.

### Mass removal measurements with a micro-analytical balance

**Fig. 3:**
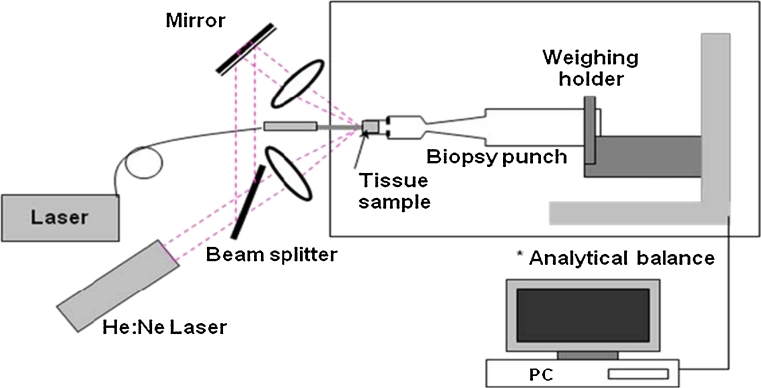
Schematic of experimental setup for mass loss measurements. The fatty tissue specimens were placed within the shallow circular well that exists at the cutting end of skin biopsy punch. The biopsy punches were attached to a weighing holder on the weight sensing wall of the micro-analytical balance. The laser energy was delivered across a short air gap into the fatty tissue. As the fatty tissue changed phase (liquefied), lipid droplets spontaneously fell away from the specimen holder to the balance surface below. The resulting changes in tissue mass were immediately observed by the micro-analytical balance measurements and recorded on a personal computer. Since the mass loss measurements require correction for tissue dehydration by evaporation, linear regression was applied to the slope of mass loss after laser irradiation to determine the corrected mass loss [13]

A micro-analytical balance (XS204, Mettler-Toledo GmbH, Greifensee, Switzerland) with a resolution of 10 μg was used for adipose tissue ablation (removal) measurements. A pair of diode laser beams (*λ* = 635 nm, S1FC635, Thorlabs, Inc., Newton, NJ, USA) were aligned to intersect at the Nd:YAG laser beam position and were used to locate the target plane for precise placement of each fat tissue specimen. Each specimen was placed in a 4-mm diameter biopsy punch mounted on the pan of the micro-analytical balance as depicted in Fig. 3. The balance was connected through a USB port to a personal computer. The tissue mass loss was recorded in real time at a rate of 5 Hz and stored in a hard disk for future analysis. Exact fat tissue mass loss from the laser irradiation was calculated by the difference between the total mass loss and the mass loss from water evaporation by the ambient air circumstance. The linear regression was applied to the slope of mass loss after laser irradiation to determine the corrected mass loss [13].

### Thermal diffusion measurements with a thermal video imaging system

Fresh porcine skin and fat was used to evaluate the thermal diffusion characteristics of 1,064, 1,320 and 1,444 nm neodymium-doped lipolysis lasers. A 600-μm silica multimode laser fiber was use to deliver laser energy to the adipose tissue for each study. The laser fibers were placed 10 mm below the skin surface, and temperature monitoring of the skin immediately above the laser irradiation area for each wavelength was performed with a thermal video imaging system (FLIR-A40M, Boston, MA, USA). Uniform lasing parameters were employed (power = 8 W, pulse energy = 200 mJ and pulse rate = 40 Hz) for each laser wavelength. Skin surface temperature was recorded in real time for each wavelength during energy delivery for 15 s at 10-ms intervals.

## Results

**Fig. 4:**
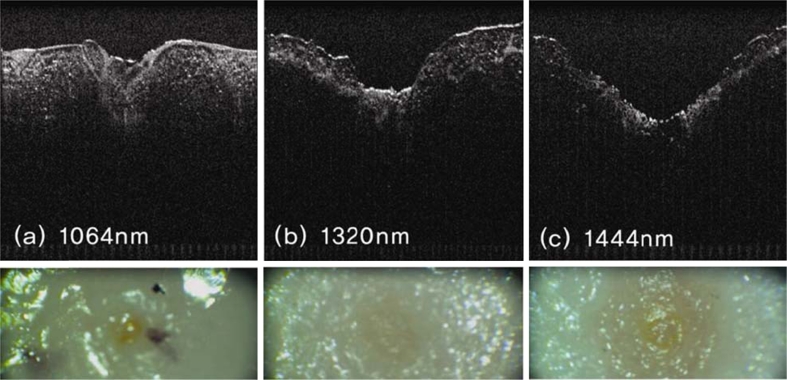
Representative OCT images (*top images*) and gross color photographic images (*bottom images*) of the craters that resulted from laser irradiation at wavelengths 1,064 (**a**), 1,320 (**b**), and 1,444 nm (**c**). These particular images resulted from laser irradiation via a 600-μm silica multimode fiber at 6 W (150 mJ pulse energy with the frequency of 40 Hz) for 10 s with a total energy delivery of 60 J

**Fig. 5:**
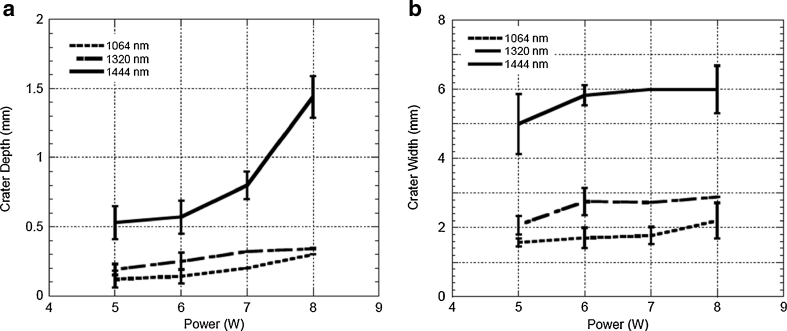
Crater size measurements (**a** depth; **b** width) after laser irradiation at wavelengths 1,064, 1,320 and 1,444 nm. Depth or width in millimeters (*y*-axis) versus laser power (Watts, *x*-axis)

The experimental design was formulated to enable quantification of tissue target effects and of mass loss from the specimens (via gravitational droplet release) as a means to compare the immediate effectiveness of lipolysis with the different wavelengths. It is possible that evaporation of water may have contributed slightly to the observed mass loss in this ex vivo experiment; however, a mathematical correction was incorporated to arrive at the exact mass loss related to lipolysis effect alone. Figure 4 presents representative OCT images from the fat tissue ablation crater at a radiant exposure of 6 W for the three different laser wavelengths. Both increased depth and width of the ablation craters was observed at 1,444 versus 1,064 or 1,320 nm. *λ* = 1,444 nm revealed the largest crater size, followed by, in order, *λ* = 1,320 and 1,064 nm. For quantitative analysis of the OCT images, measurements were taken at the deepest region of each ablation crater at each radiant exposure for the three different laser wavelengths; the crater depth and width were measured from 5 to 8 W (Fig. 5). As seen in Fig. 5a, crater depth at *λ* = 1,444 nm is increased in nearly logarithmic fashion as the incident radiant exposure increases. On the other hand, crater depth at *λ* = 1,064 and 1,320 nm remains almost monotonic over the same power interval (Fig. 5a). The quantitative results from crater size measurements after laser irradiation at three different wavelengths with ten fat tissue samples reveals significantly greater crater depth and width at all points across a range of energy settings (5– W) at 1,444 versus 1,064 or 1,320 nm (Fig. 5a, b).

**Fig. 6:**
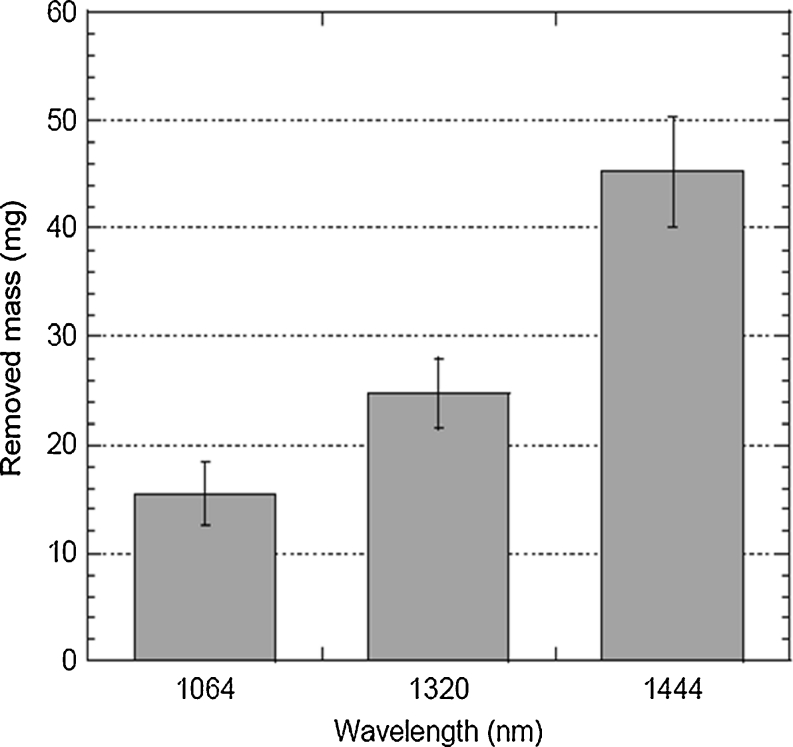
Amount of removed mass in milligrams (*y*-axis) after laser irradiation at 28.3 kJ/cm^2^ for wavelengths 1,064, 1,320, and 1,444 nm

For the quantitative evaluation of removed mass after laser irradiation, the measured mass loss for three different laser wavelengths at a radiant exposure of 28.3 kJ/cm^2^ shows that the highest mass removal for fat tissue ablation was achieved at *λ* = 1,444 nm, followed by, in order, *λ* = 1,320 and 1,064 nm (Fig. 6). As seen in Table 1, the 1,064-nm wavelength requires three times more energy than the 1,444-nm wavelength to remove the same amount of fat tissue.

**Table 1:** Quantitative values of removed mass after laser irradiation at 28.3 kJ/cm^2^ for three different laser wavelengths

	1,064 nm	1,320 nm	1,444 nm
Average mass (mg)	15.46 ± 2.92	24.78 ± 3.24	45.19 ± 5.07
Energy per gram (kJ/g)	5.17	3.23	1.77

**Fig. 7:**
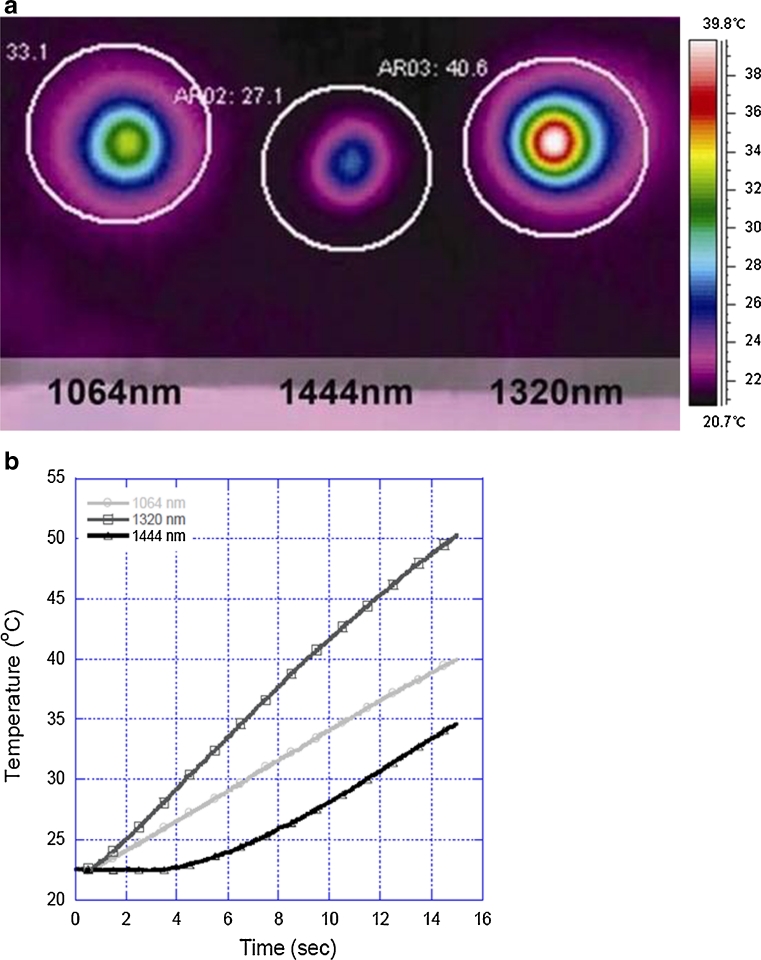
Comparison of thermal diffusion characteristics among Nd-YAG lipolysis laser wavelengths 1,064, 1,320, and 1,444 nm. **a** Thermal videographic images of ex vivo porcine skin surface temperature immediately following delivery of radiant laser energy (power 8 W, pulse energy 200 mJ, pulse rate 40 Hz, 15 s) via 600 μm silica multimode laser fiber into subcutaneous fat 10 mm below skin surface. **b** Graph depicting the rate or slope of temperature elevation during the full 15 s of energy delivery for **a** above (temperature in degrees Celsius, *y*-axis versus time in seconds, *x*-axis)

Thermal confinement directly reflects tissue absorption of radiant laser energy; increased tissue absorption improves thermal confinement and minimizes diffusion or distant spread of thermal energy. Comparison of thermal diffusion characteristics during laser irradiation of ex vivo porcine fat among the three different Nd:YAG lipolysis laser wavelengths reveals differences in both absolute temperature elevation and the rate or slope of temperature elevation as measured 10 mm from the source (tip of laser fiber) (Fig. 7). Absolute temperature elevation at the end of the recording period is greatest at 1,320 nm, intermediate at 1,064 nm, and least at 1,444 nm. The rate or slope of temperature elevation is greatest at 1,320 nm, intermediate at 1,064 nm, and least at 1,444 nm. Skin surface temperature does not increase during the initial 40 % of the recording period with 1444 nm Nd:YAG lipolysis laser irradiation.

## Discussion

Comparison studies involving 1,064, 1,320, and 1,444 nm neodymium-doped lipolysis lasers demonstrate enhanced lipolysis effect with increasing wavelength specificity for fat (Fig. 4). The increase in depth of ablation effect with increasing radiant energy exposure suggests that selective photothermolysis is at least partially responsible for lipolysis effect at 1,444 nm (Fig. 5a). Similar to 1,064 and 1,320 nm lipolysis lasers, light absorption at 1,444 nm is higher for water than for fat. However, selective photothermolysis with fat as a principal target occurs at 1,444 nm because this wavelength corresponds to an absorption peak for fat and, even though absorption for tissue water is much greater at this wavelength, fat has low water content [14].

Mathematical modeling (Monte Carlo simulations) performed for the thermal effects exerted by lipolysis lasers (1,064, 1,320, and 1,444 nm) upon adipose tissue predicts greater heat generation and a more rapid decrease in temperature with increasing tissue depth (distance from source, i.e. fiber tip) at 1,444 nm [15]. Evaluation of the thermal diffusion characteristics of 1,064, 1,320, and 1,444 nm lipolysis lasers with video thermography and external temperature monitoring is consistent with this mathematical model—thermal spread (Fig. 7) is least at 1,444 nm. These data are also consistent with relative wavelength selectivity for adipose tissue where greater energy absorption, and more localized tissue heating are expected with increasing wavelength selectivity for the intended tissue target (1,064 nm and 1,320 < 1,444 nm).

Thermal diffusivity (relative measure of heat conduction through tissue) is least at 1,444 nm, intermediate at 1,064 nm, and greatest at 1,320 nm; conversely, thermal confinement (relative indicator of heat localization near laser irradiation source or tip of laser fiber) is greatest at 1,444 nm, intermediate at 1,064 nm, and least at 1,320 nm. In some scenarios, greater thermal confinement and a corresponding limitation of the potential for unintended collateral thermal injury may be of clinical benefit during laser lipolysis [16]. Significant collateral thermal injury may not be likely during laser lipolysis-assisted body contouring where there may be large volumes of target tissue and where vital structures may be relatively distant. In contrast, the potential for undesirable collateral thermal injury is likely much higher during laser lipolysis-assisted contouring of facial structures (e.g. melolabial folds, jowls) where target tissue volumes are much smaller and where delicate structures are anatomically proximate [16].

The 1,444 nm Nd:YAG lipolysis laser accomplishes more work (mass of tissue removed) with the same amount of energy output compared to 1,064 or 1,320 nm lipolysis lasers (Fig. 6) [17]. Decreased time and/or energy required to accomplish the same amount of work indicates greater efficiency. With fat serving as tissue chromophore efficiency may be enhanced by (selective) fat liquefaction, a unique tissue-dependent mechanical phenomenon that may continuously refresh the chromophore (wherein intact fat is continuously exposed at the margin of liquefaction effect) until lipolysis is complete. Greater efficiency may also enhance safety—less energy is required by the 1,444 nm Nd:YAG lipolysis laser to accomplish a similar volume of lipolysis. Quantitatively, approximately three times more energy is required when using 1,064 nm wavelength, and two times greater energy is required when using 1,320 nm wavelength to remove the same amount of tissue (Table 1).

Because of the unique converging absorption peaks for collagen, fat, and water in the 1,400–1450 nm range, it should be recognized by clinicians that changes in tissue composition (e.g., greater collagen content) and/or the introduction of large amounts of exogenous water may likely alter the dynamics of 1,444 nm neodymium-doped laser lipolysis tissue interaction. Preliminary clinical evaluation (JDH and others) yielded further valuable insight into the nature of 1,444 nm Nd:YAG lipolysis laser tissue interaction, demonstrating rapid and effective emulsification and liquefaction of facial adipose tissue in many patients and a less robust tissue response in other patients. Since these observations have occurred among treatments in matched anatomical areas and with matched volumes of local anesthetic solution and laser treatment parameters, it is likely that the differences are explained by variation in the micro-anatomical constitution of the treated tissues—with some areas more permissive (e.g., higher adipose and lower fibrous tissue content) and other areas more refractory (e.g., lower adipose and higher fibrous tissue content) to laser lipolysis.

According to well-known principles of selective photothermolysis, greater wavelength selectivity for the intended tissue target will result in more rapid transduction of light energy to heat energy since targeted chromophores have greater ability to absorb light energy at the selected wavelength [18]. Excessive heating of unintended tissue targets may lead to non-specific collateral thermal injury or residual tissue damage (RTD). With cutaneous applications, excessive RTD may lead to delayed healing, prolonged erythema, delayed hypopigmentation, or formation of hypertrophic scar tissue [19]. In contrast, excessive RTD resulting from laser lipolysis applications may result in significant dermatologic or deep tissue injury. Laser lipolysis-associated RTD may result in full-thickness cutaneous thermal injury and subsequent necrosis requiring wound care (e.g., debridement) and secondary reconstruction.

Significant complications related to excessive RTD may occur with any lipolysis laser, especially with overly aggressive laser treatment parameters (e.g., excess energy density or amount of energy applied per unit volume per unit time), poor technique or failure to facilitate thermal confinement with sufficient local anesthetic/tumescent solution. If the treated tissue does not change phase (e.g., fat does not liquefy), then an increase in heat energy per unit volume occurs and leads to an increase in temperature [20]. The relative amount of energy and the time required to significantly damage various tissues (temperature elevation over a specific time interval) has been calculated and is described by the Arrhenius model of tissue damage due to hyperthermia [21]. Hyperthermia, though generally considered to be least severe in the continuum of thermal effects (preceding coagulation, carbonization, and ultimately vaporization) observed with heating of biological tissues, in fact may result in irreversible tissue injury.

Factors that affect degree and extent of tissue damage include radiant energy, tissue volume exposed, and duration of exposure [22]. While not an exact guide to avoidance of complications, such models help to illustrate the relative ease with which serious adverse events may occur. Irreversible tissue injury (coagulation) may occur with temperature elevation to 60 °C for only 6 s, while skin is even more susceptible with coagulation likely in as little as 1 s at 59 °C [20]). Such adverse events may be more likely with laser wavelengths with relatively less tissue specificity and thermal confinement.

With enhanced targeting of the most abundant chromophores (adipocyte lipid content, collagen, and water) the micropulsed (100 μs) 1,444 nm neodymium-doped lipolysis laser exhibits greater thermal confinement and reduced thermal diffusivity compared to earlier generation lipolysis lasers; along with superior efficiency and a corresponding decrease in total energy requirement, non-specific hyperthermia, and collateral thermal damage should also be relatively less with the micropulsed (100 μs) 1,444 nm neodymium-doped lipolysis laser. While the authors recognize certain limitations in data relevance across species, in practice, the thermodynamic characteristics of the micropulsed (100 μs) 1,444 nm Nd:YAG laser have enabled safe targeting of small volumes of fat in areas where tissues are delicate and the likelihood of undesirable secondary collateral thermal injury might otherwise be high (eg, facial areas including mid- and lower face) [16].

## Conclusion

The wavelength dependence measurements of laser lipolysis effect were performed using different lasers at 1,064, 1,320, and 1,444 nm wavelengths that are currently used in the clinic. The observations reported in the study demonstrate that the greatest ablation crater size and mass removal in fatty tissue was achieved at the 1,444 nm wavelength followed by, in order, 1,320 and 1,064 nm. In the evaluation of heat distribution at different wavelengths, enhanced thermal confinement was achieved at the 1,444 nm wavelength. This study shows that fat ablation is wavelength dependent, with *λ* = 1,444 nm providing the largest craters and greatest amount of mass removed at all radiant exposures. Therefore, *λ* = 1,444 nm provided the highest ablation efficiency as compared with the other two laser wavelengths, *λ* = 1,064 and 1,320 nm.
